# Peptide-Fluorescent Bacteria Complex as Luminescent Reagents for Cancer Diagnosis

**DOI:** 10.1371/journal.pone.0054467

**Published:** 2013-01-18

**Authors:** Bing Dong, Anxin Wang, Lihua Yuan, Lisha Chen, Kefeng Pu, Wei Duan, Xiyun Yan, Yimin Zhu

**Affiliations:** 1 Suzhou Key Laboratory of Nanobiomedicine, Division of Nanobiomedicine, Suzhou Institute of Nano-Tech and Nano-Bionics, Chinese Academy of Sciences, Suzhou, Jiangsu, China; 2 National Laboratory of Biomacromolecules, Institute of Biophysics, Chinese Academy of Science, Beijing, China; 3 University of Chinese Academy of Sciences, Beijing, China; 4 School of Medicine, Deakin University, VIC, Australia; University College London, United Kingdom

## Abstract

Currently in clinic, people use hematoxylin and eosin stain (H&E stain) and immunohistochemistry methods to identify the generation and genre of cancers for human pathological samples. Since these methods are inaccurate and time consuming, developing a rapid and accurate method to detect cancer is urgently demanded. In our study, binding peptides for lung cancer cell line A549 were identified using bacteria surface display method. With those binding peptides for A549 cells on the surface, the fluorescent bacteria (*Escherichia coli* with stably expressed green fluorescent protein) were served as specific detecting reagents for the diagnosis of cancers. The binding activity of peptide-fluorescent bacteria complex was confirmed by detached cancer cells, attached cancer cells and mice tumor xenograft samples. A unique fixation method was developed for peptide-bacteria complex in order to make this complex more feasible for the clinic use. This peptide-fluorescent bacteria complex has great potential to become a new diagnostic tool for clinical application.

## Introduction

With the development of new techniques regarding diagnosis and treatment for cancers, the mortality rate of cancer patients has decreased in the past decades. However, the aim to cure cancers is still far from reaching. Currently, the key points to increase the cure rate and life quality of cancer patients are earlier and accurate diagnosis and effective treatment for such malignant diseases. The application of sensitive luminescent reagents and targeting molecules for cancer cells provides useful tools for accurate diagnosis and effective treatment for cancers. Novel luminescent materials such as quantum dots [1], upconversion nanomaterials [2] and nanobubbles [3] have been attempted to apply to the cancer imaging systems. Various molecules such as antibodies [4], peptides [5], [6] and aptamers [7] have been used as targeting molecules for cancer diagnosis and therapy. Although there are some progress for the development of luminescent materials and targeting molecules, effective systems for cancer diagnosis and therapy have not been fully established. Earlier and more accurate diagnosis methods combining targeting molecules with robust imaging molecules attract more and more researchers’ interest [8]. Our study intends to establish a novel system which includes targeting peptides and fluorescent bacteria for the accurate diagnosis of cancers. Targeting peptides could be screened and identified by bacteria surface display method developed in the 1990s [9], [10]. Using the fluorescence activated cell sorter (FACS), various peptides with specific binding activity have been obtained in a high throughput way with bacteria display method [11], [12]. Currently, with this method, the targeting peptides for breast cancer cells [13] and substrate peptides for proteinase [14] have been identified. In our study, with the identification of the specific binding peptides for lung cancer A549 cells, a new technique for detecting lung cancer cells using peptide-fluorescent bacteria system would be established. Furthermore, peptide-fluorescent bacteria system could be used as the diagnostic reagent in clinical application for cancer patients in future.

## Materials and Methods

### 1. Cell Culture and Materials

Human lung carcinoma cell lines (A549 cells and H460 cells), human breast adenocarcinoma cell line (MCF-7), human hepatocellular carcinoma cell line (HepG-2), human cervical carcinoma cell line (HeLa) and human laryngeal carcinoma cell line (Hep-2) were used in this study. These cell lines were grown in RPMI-1640 (Hyclone Corp., USA) and human lung fibroblast cells (HLF) were cultured in Dulbecco’s Modified Eagle Medium (Hyclone Corp., USA) in a 5% CO_2_ humidified incubator at 37°C. The medium was supplemented with 10% fetal bovine serum (FBS) (Hyclone Corp., USA) and 1% penicillin/streptomycin (China National Medicine Corp., China). A549, H460 and HLF cells were kind gifts from Dr Biliang Zhang [15], [16] (Guangzhou institutes of biomedicine and health, CAS, China). MCF-7, HepG-2, HeLa and Hep-2 cells were kind gifts from Dr Haiyan Liu [17], [18] (The Cyrus Tang Hematology Center, Soochow University, China). The other chemical agents in this study were purchased from the China National Medicine Corporation.

### 2. Screening of Binding Monoclonal Peptide-fluorescent Bacteria with A549 cells

The bacterial peptide library of *E. coli* used in this study was a gift from Patrick S. Daugherty in the Department of Chemical Engineering, University of California, Santa Barbara. In this bacterial peptide library, every *E. coli* surface presented 13-mer peptide (X2CX7CX2) fusing in the second extracellular loop of the circularly permuted outer membrane protein OmpX (CPX) [13], in which expressed peptides and green fluorescent protein (GFP) were induced by the addition of L-(+)-arabinose (0.02% w/v) to a log-phase culture [13], [19].

Frozen aliquots of 1×10^9^ bacteria were thawed and cultivated overnight in super optimal broth (SOB) at 37°C and supplemented with 34 µg/ml chloramphenicol (CM) and 0.2% (w/v) D-(+)-glucose. The next day, bacteria were subcultured at 1∶50 in Lysogeny broth (LB) medium supplemented with 34 µg/ml CM for 2 h at 37°C and induced by 0.02% (w/v) L-(+)-arabinose for 1 h at room temperature to ensure GFP and peptides expressed in bacteria. After cultured (48 h post-seeding) A549 and HLF cells were harvested by trypsinisation and resuspended in tubes. 10^7^ HLF cells were co-incubated with 100-fold excess bacterial cells for 45 min on an inversion shaker at 4°C. Cell suspensions were centrifuged at 1000 rpm for 5 min at 4°C and the supernatant was collected. This procedure might be repeated for another time due to the increased non-specific binding events occurred for HLF cells. The supernatant was co-incubated with 10^7^ A549 cells for 45 min on an inversion shaker at 4°C and cell suspensions were centrifuged at 1000 rpm for 5 min at 4°C and then washed three times with PBS. The sediment was resuspended with 5 ml cold PBS and immediately analyzed by FACS (BD FACS Aria II, BD Corp. USA). The sorting gate in the FACS experiment was set based on the fluorescent signals of negative control samples. Once the fluorescence settings had been optimized for the negative control, an appropriate sort gate was drawn for sorting the library based on fluorescence. A sort gate was drawn to exclude as much of the negative control as possible while still capturing positive events in the library [13].The green fluorescent cells were gated for sorting because if peptides which were on the surface of green fluorescent bacteria could bind with A549 cells and the green fluorescent signals of cells would increase. At least 10^5^ events were recorded and the tumor cells with increased green fluorescent signals and dropped into the orange gate were collected with FACS. The collected cells were cultured overnight in SOB containing 0.2% D-(+)-glucose and 34 µg/ml CM. Bacteria binding with cancer cells specifically were amplified for the next round of screening. From the next round, 10^6^ cells were enough for FACS analysis and sorting. Until the fluorescent signal of tumor cells stop increasing in sequentially two rounds, procedure of screening for binding peptides was terminated. Selected bacteria with binding peptides were cultured on the LB medium plate containing 1% agar and 34 µg/ml CM. After cultured for 24 h at 37°C, monoclonal peptide-fluorescent bacteria were picked and cultured in 5 ml SOB containing 0.2% (m/v) D-(+)-glucose and 34 µg/ml CM at 37°C overnight. The next day, the binding activity of individual clones was examined by measuring the GFP fluorescent signals of cells after A549 cells incubated with those monoclonal peptide-fluorescent bacteria. The sequences of binding peptides on the surface of monoclonal peptide-fluorescent bacteria were obtained by sending out those bacteria to Sangon Biotech Co., Ltd. Shanghai, China for sequencing.

### 3. Characterization of Binding Specificity and the Efficiency between Peptide-fluorescent Bacteria and Cells

The experiments were performed on both attached and detached cells. The fluorescence microscopy experiment was firstly carried out to examine the binding specificity of monoclonal peptide-fluorescent bacteria with attached cells. A549 cells, HLF cells, H460 cells, MCF-7 cells, Hep-2 cells, HepG-2 cells and HeLa cells (3×10^5^) were plated in 12-well cell culture plates one day before the experiment. After induction, 100 µl of bacteria suspension(≈8×10^7^) in 1 ml of PBS were incubated with all kinds of cells at room temperature which has been proved to ensure proteins on the surface of cells expressed normally during the incubation [13] for 45 min and were washed three times by PBS. Cells were photographed with fluorescence microscopy (Nikon Ti, Nikon Corp. Japan) to confirm the binding activity of monoclonal peptide-fluorescent bacteria with cells. The binding activity between monoclonal peptide-fluorescent bacteria with detached cells was examined by flow cytometry. The procedure of examining the binding activity of peptide-fluorescent bacteria is similar to that of screening the binding peptides with cells.

### 4. Attempt of Maintaining Bacteria Binding Activity with Various Fixation Methods

Fixation was done with different fixation reagents (ethanol [20], methanol [21], acetone [22] and 4% paraformaldehyde [23]). After centrifuged at 5000 rpm for 5 min, the supernatant was removed and the bacteria were fixed in the fixation reagents for 20 min at room temperature. The excess fixation reagents were removed and the fixed bacteria were suspended by PBS and stored at 4°C.

Flow cytometry analysis and fluorescence microscopy experiments were performed as indicated previously to examine the binding activity of fixed bacteria with cells. Compared with fresh monoclonal peptide-fluorescent bacteria, the ratio of fixed bacteria to cells varied from 100∶1 to 500∶1 to obtain obvious and detected fluorescent signals in cells. Normally, after bacteria were fixed, the green fluorescent signals of bacteria decreased at certain extent. To examining whether the binding activity of fixed bacteria with cells was depended on time, fluorescence microscopy and FACS analyses were carried out repeatedly at day 1, day 7 and day 30 after fixation.

### 5. Examination of Binding Activity of Peptide-fluorescent Bacteria System with Tumor Tissue of Mice Xenograft

Binding experiment was performed on paraffin-embedded tissue. A549 mice tumor xenograft samples on paraffin slides were kind gifts from Dr Laura Cerchia and Dr Vittorio De Franciscis (Istituto di Endocrinologia ed Oncologia Sperimentale, CNR, Naples, Italy). The related paper about samples preparation has already been published in the PLos One journal [24]. The dewaxing procedure for the paraffin samples was done following the accepted procedure [25]. 1×10^6^ (in 100 µl) fresh peptide-bacteria and 5×10^6^ fixed peptide-bacteria were incubated separately with paraffin tumor sections for 1 h at room temperature. After washing with PBS three times, tumor sections were observed under a Nikon A1R confocal laser scanning microscope (Nikon Corp. Japan).

## Results

### 1. Enrichment of Specific Binding Peptides Library for A549 cells

**Figure 1:**
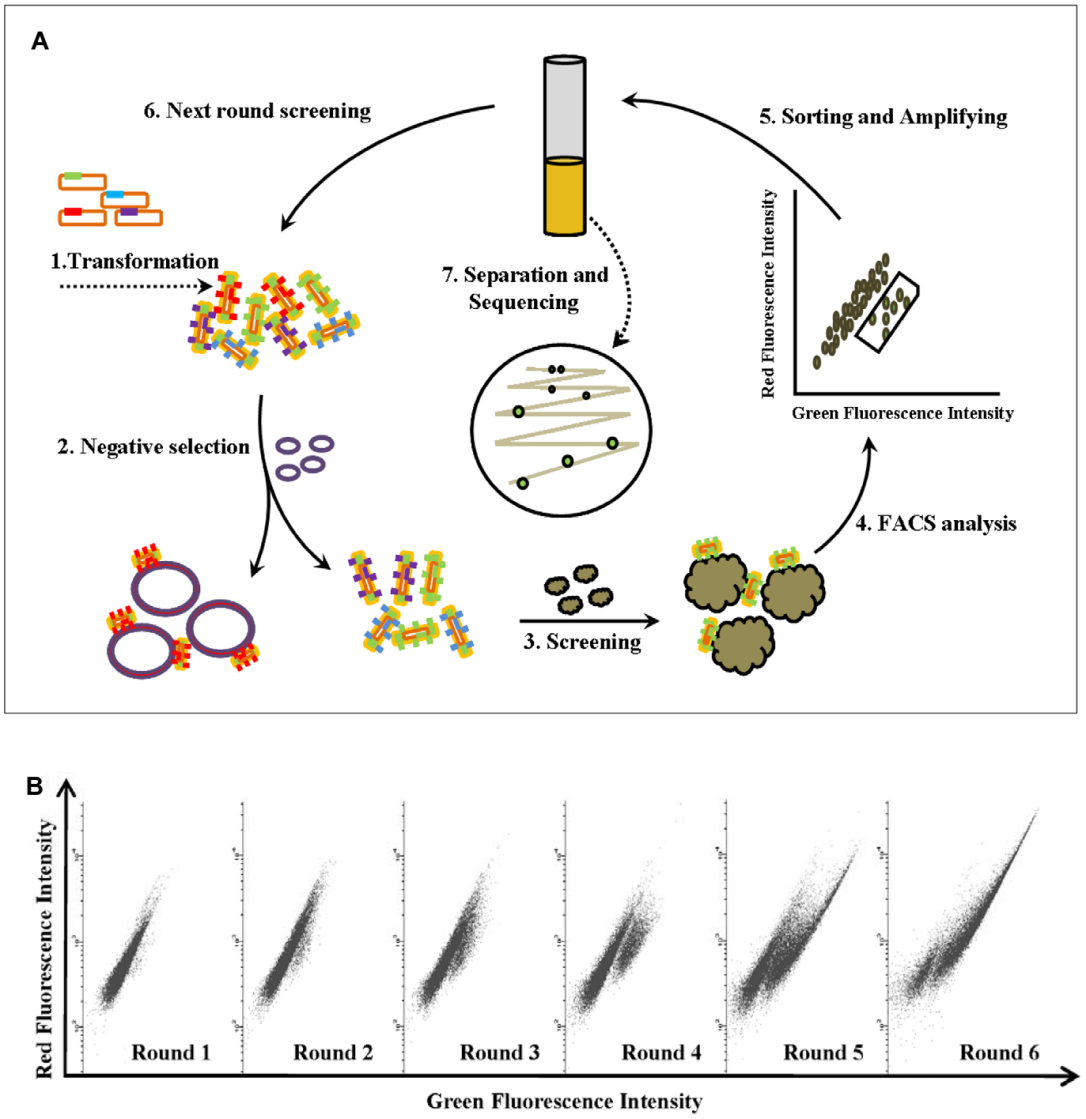
The process of screening and enriching the binding peptide library for cancer cells with bacteria surface display methods. (**A**) Schematic representation of peptides screening and selection with bacterial display library. 1. Constructed the library by transforming plasmids into *E.coli* MC1061; 2. Discarded the bacteria binding with normal cells after pre-incubation; 3. Incubated the mixture of cancer cells and residual bacteria; 4. Analyzed the binding effect of cancer cells with bacteria using FACS; 5. Sorted the binding bacteria to cancer cells by flow cytometry and cultured the binding bacteria in medium; 6. Performed the next round binding bacteria screening; 7. Isolated the binding bacteria to cancer cells and sequenced the peptides displayed on the surface of the bacteria. (**B**) The progress of enrichment of bacteria binding with cancer cells by FACS in 6 screening rounds.

In order to isolate cell specific binding ligands for a given tumor cell phenotype we decided to set up a model system including normal (non-tumor) cell line (HLF) and lung carcinoma cell line (A549). HLF cells worked for counterselection in screening process. A 13-mer library with cysteine restrained peptides was utilized for selection of binding peptides against intact cells (Figure 1A). At each round of A549 cells selection step one or two counterselection steps against HLF cells were performed. During the selection process, we progressively increased the selective pressure by changing incubation condition, washing condition and area of gate in FACS. During round 6 and 7, the ratio of green fluorescent cells to whole cells remained unchanged, suggesting that the population had stopped to evolve under the selection pressure. Indeed, the pool at round 6 was enriched for peptides displayed on the surface of bacteria that preferentially bind to A549 cells (Figure 1B). Sixty individual clones of bacteria from this pool were chosen and grown for the next step of analysis.

### 2. Identification of Monoclonal Peptide-fluorescent Bacteria with High Binding Efficiency

**Figure 2:**
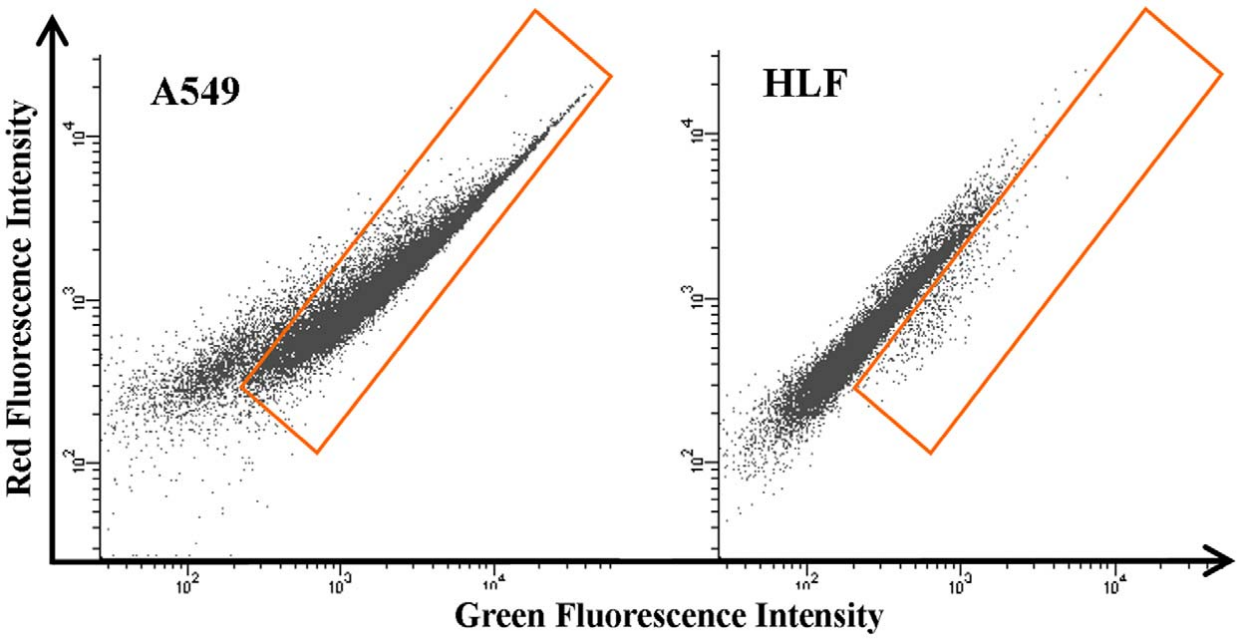
FACS results of selected monoclonal peptide-fluorescent bacteria binding with A549 cells and HLF cells. The binding fraction of A549 cells with bacteria (show in the orange gate) was 80% and that of HLF cells was 0.4%.

The monoclonal peptide-fluorescent bacteria with high binding efficiency were identified from 60 clones for further experiments. Before the clones were sent for sequencing, we used FACS to compare the binding efficiency of bacteria to HLF and A549 cells. After incubation with induced monoclonal peptide-fluorescent bacteria, the percentage of the fluorescent cells to whole cells represented the binding rate of peptides on the surface of clonal bacteria with cells. The results indicated that the binding rate of all the bacteria from those 60 clones was around 10%–80% for the A549 cells, while it was less than 5% for HLF cells (data not shown). Comparing to those with 20% binding rate, peptides-fluorescent bacteria with 80% binding rate might have higher affinity to cells. The binding rate of the monoclonal peptide-fluorescent bacteria (clone 4) with the highest binding efficiency was 80% for A549 cells and 0.4% for HLF cells (Figure 2). The sequencing results showed that seven unique clone sequences were obtained from 60 clones (Table 1). There was no conserved sequence observed for all these clones.

**Table 1:** Sequences of peptides.

	Sequence of peptides	Binding Rate	Frequency
1	WFCSWYGGDTCVQ	80%	42
2	WKCTKYVCVL	40%	11
3	LNCSTKSLGYCVW	36%	2
4	WMCMTSPSRMCRN	10%	2
5	GYCEHTRMMLCQF	41%	1
6	GRCRLNGDCYCVR	9%	1
7	NSCTREQGVECGN	8%	1

Sequences and binding rates of different peptides with A549 cells. Peptides were ordered by frequency in experiments.

### 3. Evaluation of the Binding Specificity of the Monoclonal Peptide-fluorescent Bacteria

**Figure 3:**
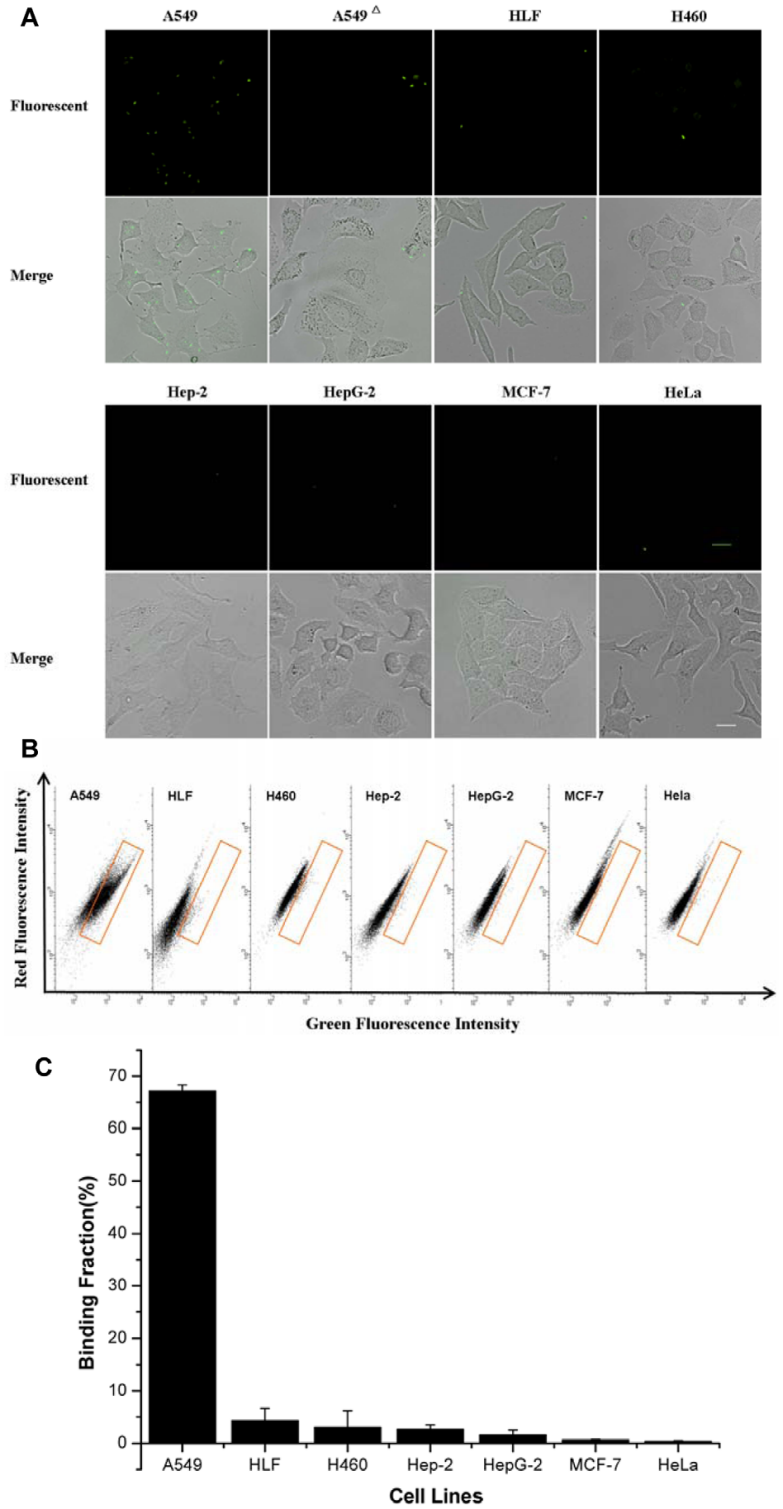
The results of selected monoclonal peptide-fluorescent bacteria specifically binding with various cells. (**A**) Fluorescence microscope images of bacteria clones incubated with cells. A549**^△^** was incubated with CPX only bacteria and other cells were incubated with selected monoclonal peptide-fluorescent bacteria, the scale bar was 20 µm. (**B**) FACS results of various cells binding with monoclonal peptide-fluorescent bacteria at ratio 1∶100. (**C**) Percentage of binding fraction of monoclonal peptide-fluorescent bacteria with various cells from FACS data. Data were mean ± S.D. of at least three independent experiments.

The identification of a small set of peptide-fluorescent bacteria that may distinguish the A549 cells from the HLF cells raises a question of whether these peptide-fluorescent bacteria may bind as well with other cell types. For this aim we determined to examine the relative binding potential of all the peptide-fluorescent bacteria to several cell lines. We first determined the cell type specificity by measuring the binding activity of all the peptide-fluorescent bacteria on a panel of unrelated cell lines. We found that any of the seven peptide-fluorescent bacteria did not bind to other human carcinoma cell types including liver (HepG-2), laryngeal (Hep-2), cervical (Hela) and breast (MCF-7) carcinoma cells. The results from the observation of fluorescent microscope and flow cytometer on attached and detached cells verified the binding specificity of the peptide-fluorescent bacteria. Figure 3 showed the typical results of the peptide-fluorescent bacteria which had the highest binding efficiency to A549 cells (clone 4). Furthermore, the bacteria with only the CPX scaffold protein could not bind with A549 cells, which meant peptides presenting on the surface of the bacteria mediated the binding event between bacteria and A549 cells. More attention needs to be paid to other lung carcinoma cells e.g., H460. Our results illustrated that the monoclonal peptide-fluorescent bacteria could not recognize H460 cells –another cell line also belonging to the category of non-small-cell lung cancer, which implied that the monoclonal peptide-fluorescent bacteria have the possibility of recognizing different genres of lung cancer cells.

### 4. Maintenance of Binding Ability of Peptide-fluorescent Bacteria with Tumor Cells for Clinical Application

**Figure 4:**
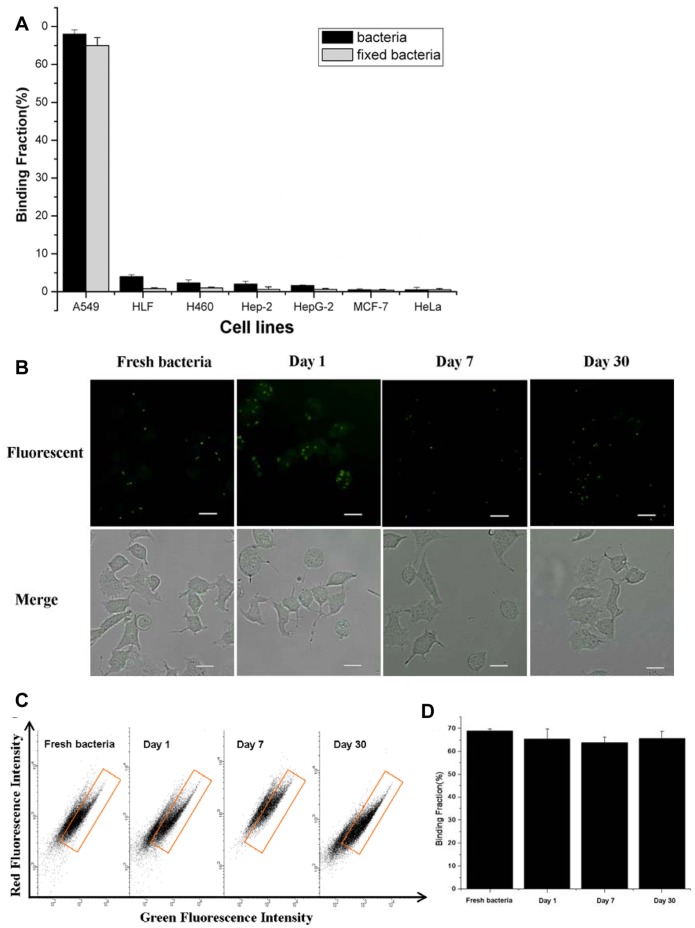
Characterization of binding ability of fixed bacteria. (**A**) Percentage of binding fraction of fresh and fixed monoclonal peptide-fluorescent bacteria with various cells from FACS data. Cells binding to fresh bacteria (black) at ratio 1∶100 and binding to fixed bacteria (gray) at ratio 1∶500. (**B**) The fluorescence microscope images of A549 cells incubating with fresh bacteria and fixed bacteria at ratio 1∶500, at day 1, day 7 and day 30 after fixation and the scale bar was 20 µm. (**C**) FACS results of A549 cells binding with fresh and fixed monoclonal peptide-fluorescent bacteria at ratio 1∶500 at different time points. (**D**) Percentage of binding fraction of fresh and fixed monoclonal peptide-fluorescent bacteria with A549 cells from FACS data Cells binding with fresh bacteria and fixed bacteria at ratio 1∶500 at different time points. Data were mean ± S.D. of at least three independent experiments.

One fixation method was needed to maintain the binding specificity and efficiency of bacteria to cells for more extensive applications. Through comparing the binding effect of bacteria with cells after bacteria were fixed by ethanol, methanol, acetone and 4% paraformaldehyde, we chose paraformaldehyde as the fixation reagent for further study. Although the fixed bacteria still kept the ability to bind with cells, the binding efficiency of fixed bacteria was lower than that of fresh bacteria. For obtaining a comparable binding effect for the fixed bacteria, we increased the ratio of bacteria to cells from 100∶1 to 500∶1. The results shown in Figure 4A and S1 indicated that fixed bacteria still maintained the binding specificity. Moreover, after comparison with negative control cells, non-tumor cell line (HLF) with some tumor cell lines (H460 and Hep-2), the binding specificity of fixed bacteria was better than that of fresh bacteria. Subsequently, we examined the change on the binding efficiency of fixed bacteria following the time lapse. In this experiment, the objectives we chose were fresh bacteria, freshly fixed bacteria (day 1) and fixed bacteria stored for 7 days and 30 days. The results (Figure 4B, 4C and 4D) showed that the fixed bacteria still kept a higher binding efficiency with cells even after being stored for 30 days, which indicated that fixed fluorescent bacteria could be used to detect A549 cells even after being stored for quite a long time.

### 5. Detection of A549 Tumor Tissue from the mice Xenograft with Peptide-fluorescent Bacteria

**Figure 5:**
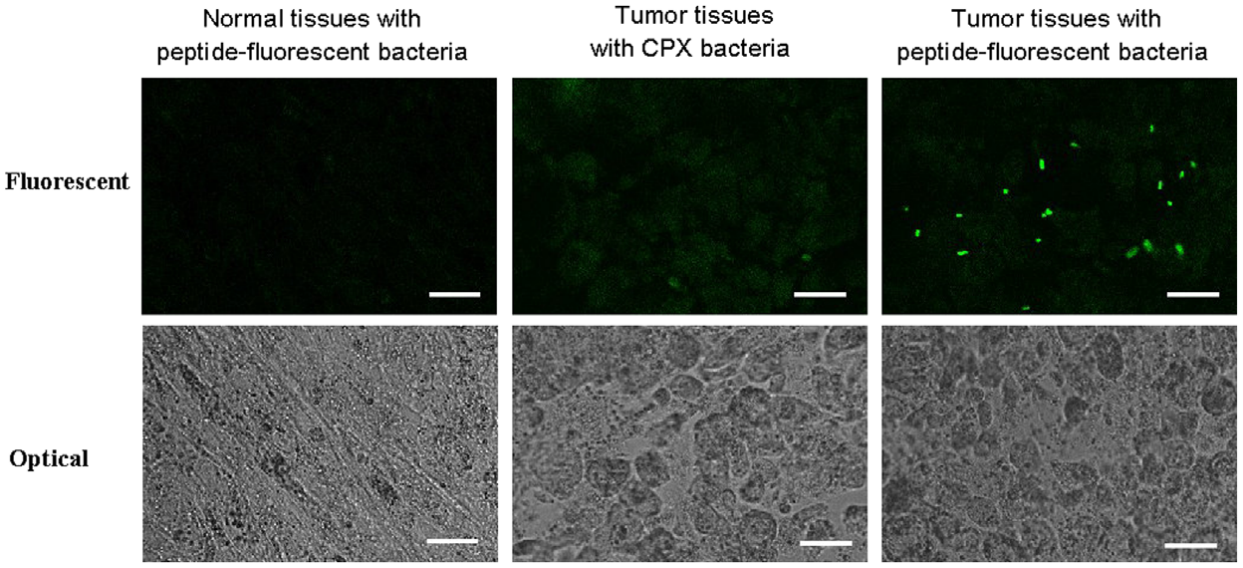
The representative images of selected peptide-fluorescent bacteria binding with tissue sections from the mice xenograft. The scale bar was 20 µm. Data were representative of at least three independent experiments.

After incubated tumor tissue in the A549 mice xenograft with induced monoclonal peptide-fluorescent bacteria (clone 4 and CPX only), tumor section in the xenograft emitted green fluorescence because peptides on the surface of fluorescent bacteria could recognize the tumor cells. However, tissue section adjacent to the tumor part incubated with clone 4 peptide-bacteria and tumor section incubated with CPX only bacteria did not emit fluorescence signals (Figure 5). These results could be reproduced with fixed peptide-fluorescent bacteria (data not shown).

## Discussion

In our study, specific binding peptides for lung cancer cells were identified with bacteria surface display method. There were no conserved sequences for those peptides which indicated that those peptides might interact with different proteins on the surface of tumor cells. Although those peptides might be used as target molecules for diagnosis and treatment for cancers, in this study, we tried to explore the potential of fluorescent *E. coli* MC1061, which had specific peptides on the surface, directly worked as diagnostic reagents. The results from the *in vitro* experiment indicated that the selected bacteria with peptides on the surface could specifically bind with A549 cells with high affinity, regardless if the cells were attached or detached. The ability of peptide-fluorescent bacteria to recognize certain tumor cells was confirmed by mice tumor xenograft experiments. With the results from the *in vitro* experiments, a novel method was developed for the first time to detect lung cancer cells directly with fluorescent bacteria. Considering the shelf time and safety of bacteria, manipulation of live bacteria was necessary for their further application. Through comparing the effect of different fixation reagents (ethanol, methanol, acetone and 4% paraformaldehyde), 4% paraformaldehyde was selected as the best fixation reagent. The bacteria fixed with this reagent could maintain a high binding efficiency and specificity to cells for at least one month.

In the clinical practice and bench work, most of time, imaging technology was used for diagnosis of lung cancer. Many imaging molecules already have clinical and sub-clinical usage, for examples, functional radioactive molecules for PET imaging [26], novel magnetic materials for MRI imaging [27], new materials for ultrasonic imaging [28] and PET/MRI imaging [29]. Even there are already reports about the bacterium as a bioluminescent imaging reagent in diagnosis [30], but its application was limited due to the scarcity of the precision, sensitivity and luminescent strength of this imaging reagent. Our new system can help us not only to discriminate lung cancer cells from other cells, but also to determine the genre of lung cancer cells, which makes this method more accurate for diagnosis. At present, methods that are used clinically to determine the genre of cells include Hematoxylin and eosin stain (H&E stain) [31] and immunohistochemistry [32], but these two methods are more complicated, costly and subjective compared with our new method. With low investment (only one fluorescent microscope needed) and short preparation time of samples, our method provides a new supplementary way for making rapid and accurate decisions about whether tumors are benign or malignant, where is the edge of tumor section and even what genre of tumor the patient has during surgical operations [33], [34].

Our study presented a new method to detect lung cancer cells directly with peptide-fluorescent bacteria as luminescent reagents *in vitro*. This method has the advantages of low cost, ease of procurement, ease of performance, short time preparation and objectivity. However, currently we could not elucidate the detailed molecular mechanism about the binding event between peptides and cells. Moreover, we still need more clinical samples to confirm the validity of our system. Even though, from the present experimental results, we believe that this method has huge potential as a new clinical diagnostic means. In future study, support from mass experimental data is needed to promote the translation of this method from the bench to the clinic.

## Supporting Information

Figure S1 **FACS results of various cells binding with fixed monoclonal peptide-fluorescent bacteria at ratio 1∶500.**
(TIF)
