# Supplementary figures and images for: Peptide-Fluorescent Bacteria Complex as Luminescent Reagents for Cancer Diagnosis
Source: PLoS One. 2013 Jan 18;8(1):e54467. doi: 10.1371/journal.pone.0054467 (PMC3548802; doi:10.1371/journal.pone.0054467)

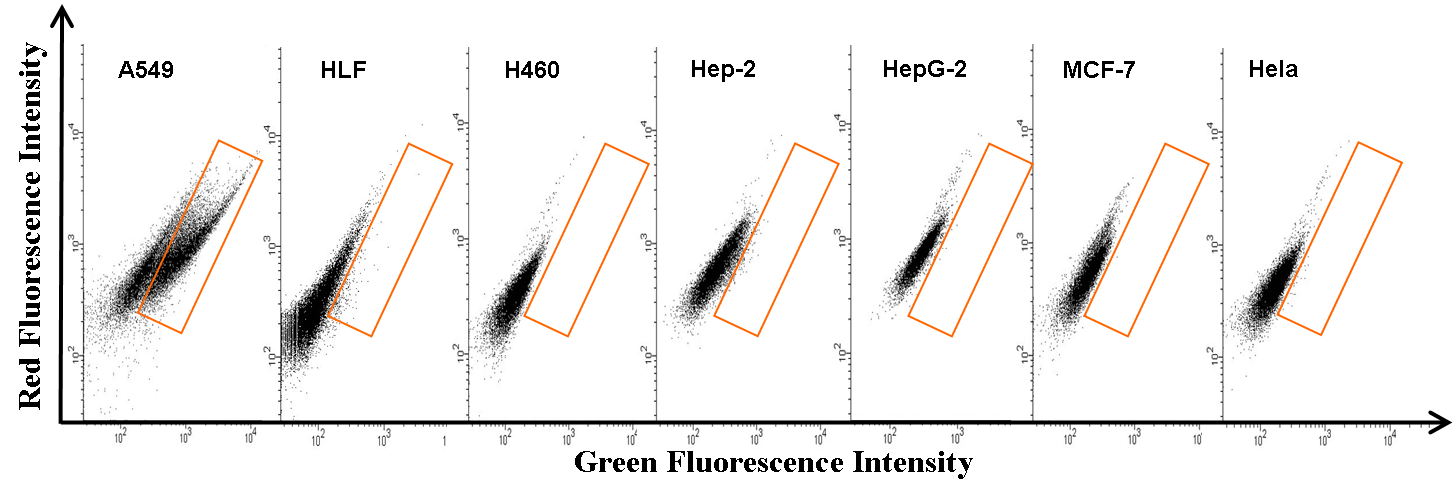

Supplement: Figure S1 — FACS results of various cells binding with fixed monoclonal peptide-fluorescent bacteria at ratio 1∶500. (TIF) [file pone.0054467.s001.tif]
